# HistomicsTK: A Python toolkit for pathology image analysis algorithms

**DOI:** 10.1016/j.softx.2025.102318

**Published:** 2025-08-22

**Authors:** Fattaneh Pourakpour, Ákos Szölgyén, Ramin Nateghi, David A Gutman, David Manthey, Lee AD Cooper

**Affiliations:** a Department of Electrical Engineering, McCormick School of Engineering, Northwestern University, USA; b Department of Pathology, Feinberg School of Medicine, Northwestern University, USA; c Department of Urology, Feinberg School of Medicine, Northwestern University, USA; d Department of Pathology, Emory School of Medicine, Emory University, USA; e Kitware, Inc., USA; f Chan Zuckerberg Chicago Biohub, USA

**Keywords:** Image analysis, Medical imaging, Digital pathology, Pathology, Microscopy

## Abstract

Growth in the digital imaging of glass tissue slides has produced petabytes of data, however, this data remains underutilized in biomedical research due in part to a lack of open-source software. HistomicsTK is an open-source Python package that provides preprocessing, segmentation, and feature extraction capabilities for building histology image processing pipelines. HistomicsTK can function as a standalone Python package or serve containerized pipelines through a web-based interface using the Digital Slide Archive platform. This paper provides an overview of HistomicsTK with illustrative use cases and describes how this project engages the community in software development and maintenance.

## Metadata

**Table:** 

Nr	Code metadata description	
C1	Current code version	1.4.0
C2	Permanent link to code/repository used for this code version	https://github.com/DigitalSlideArchive/HistomicsTK
C3	Permanent link to reproducible capsule	https://doi.org/10.5281/zenodo.14833780
C4	DOI for this version	https://doi.org/10.5281/zenodo.14833781
C5	Legal code license	Apache License, 2.0 (Apache-2.0)
C6	Code versioning system used	git
C7	Software code languages, tools and services used	Python
C8	Compilation requirements, operating environments and dependencies	Python ≥ 3.9
C9	If available, link to developer documentation/manual	https://digitalslidearchive.github.io/HistomicsTK/histomicstk.html
C10	Support email for questions	kitware@kitware.com

## 1. Motivation and significance

Healthcare operations and biomedical research in pathology produce millions of histopathology glass slides intended for microscopic examination each year, and increasingly, these slides are being digitized using high-throughput microscopy scanners to produce whole-slide images (WSIs) [1,2]. These digital images effectively replace the glass by capturing the entire slide at high resolution. Unlike glass, WSIs can be recalled from storage and shared instantly. They also enable the application of image analysis tools to quantify tissue patterns or to make predictions. The development of artificial intelligence (AI) and image analysis tools for WSI has become an active research area, with hundreds of studies investigating medically relevant applications like diagnosis and prognosis [3-7]. The image analysis goals for biomedical studies often focus on identifying and characterizing tissue structures rather than making patient-level predictions. This often entails tasks like image segmentation and the extraction of quantitative morphologic features. In biomedical research, however, WSI analysis remains underutilized due to few accessible, comprehensive software tools that can handle the computational challenges of processing large-scale histopathology images.

The adoption of genomics in biomedical research has benefitted from the availability of mature, open-source software tools for genomic data analysis. These tools are complementary and interoperable using community-developed standards. In contrast, digital pathology, another data-intensive domain in biomedical research, faces a less cohesive open-source ecosystem and is more fragmented, with tools narrowly focused on specific tasks and often lacking interoperability. This presents challenges to biomedical researchers, who must string together different software tools to construct complete image analysis pipelines, which requires more than a basic understanding of image analysis methods. Additionally, the wide variety of WSI formats, including many proprietary ones still used by vendors, further complicates analysis workflows. Furthermore, open-source tools primarily focus on patient-level predictions that are relevant to clinical applications, rather than addressing the analytical needs of biomedical researchers. While full-featured commercial image analysis tools exist, they are often expensive and, by and large, are not customizable or extensible. Commercial tools also often have a pricing structure that increases costs for “unlocking” compute parallelism, or their licensing may prohibit installation on academic computing clusters, limiting the volumes of data that they can analyze.

We developed HistomicsTK as an open-source histology image analysis library for the biomedical research community. HistomicsTK was developed as part of a software ecosystem that includes the Digital Slide Archive (DSA) [8], a centralized, web-accessible platform for managing and sharing WSI datasets, and HistomicsUI, a specialized interface for image annotation and analysis. HistomicsTK can serve as a standalone Python package for constructing image analysis pipelines or as a DSA plugin to serve pipelines to biomedical investigators over the web. HistomicsTK has been successfully employed in several significant research initiatives, including quantitative analysis of tumor-infiltrating lymphocytes (TILs) in breast cancer [9,10], and the extraction of pathomic features from hematoxylin and eosin (H&E) stained images to predict the expression of 50 protein biomarkers [11]. Another study employed the positive pixel count (PPC) algorithm, developed within the HistomicsTK framework, to quantify fibrosis in kidney allograft biopsy specimens [12]. Similarly, another investigation utilized the same algorithm to examine the correlation between age-related cognitive impairment and neuropathological features [13]. Furthermore, HistomicsTK was used to extract a range of morphological features to establish a digital histological biomarker for the prediction of invasive breast cancer [14]. Additionally, it has been applied for the advanced classification and segmentation of nuclei in breast cancer specimens [15]. These diverse applications highlight HistomicsTK’s robust capabilities in supporting sophisticated computational pathology workflows across different research contexts and disease domains.

HistomicsTK has been widely adopted across a range of advanced analytical tools and GitHub repositories in computational pathology and genomics [16-24]. RiskFormer [16], a deep learning framework for breast cancer risk prediction from WSIs, employed HistomicsTK’s preprocessing subpackage to distinguish tissue regions from the background in brightfield H&E images. In [17], tissue area was quantified using HistomicsTK’s tissue detection algorithm by generating a binary mask and summing the corresponding pixel area. CellViT++ [18], a foundation model-based method for cell segmentation and classification, incorporated HistomicsTK as a conventional machine learning baseline for comparative performance evaluation. In [19], HistomicsTK provides color normalization, nuclei segmentation, and localization in histological images. Starfysh [20], which integrates spatial transcriptomic and histologic data, leveraged HistomicsTK’s color deconvolution for stain unmixing. Similarly, StLearn [21], a toolkit for downstream spatial transcriptomics analysis, implemented HistomicsTK’s color deconvolution and segmentation functions in its pipeline. Additionally, HistomicsTK’s color deconvolution was employed to extract the Hematoxylin stain image for subsequent cellularity ratio computation [22]. The Feature Re-calibration based Multiple Instance Learning (FRMIL) repository [23] also utilized HistomicsTK’s tissue detection capabilities for WSI classification.

### 1.1. Related work

A number of open-source tools and libraries have been developed for working with digital pathology images. Reading WSIs is a popular theme for open-source tools, with many libraries providing tiling capabilities that allow users to read a WSI as a sequence of smaller image tiles. Tools such as Histolab [25], py-wsi [26], PathEX [27], PyHIST [28], and Compay-syntax [29] fall into this category, offering various strategies for tile reading, including semi-automatic and random tile extraction, support for reading WSIs via OpenSlide [30], and compatibility with Aperio format XML annotation files [26]. Histolab [25] also supports preprocessing tasks such as tissue detection and artifact removal. HistoQC [31] is focused on quality control of WSIs, facilitating artifact detection and the identification of batch effects, such as staining intensity variations across slides. Other tools provide utilities designed to support preprocessing and image analysis: deep-histopath [32] includes nuclei scoring functionality, while CellProfiler [33] supports quantitative analysis and feature extraction from microscopy images, particularly in high-throughput experiments in cell biology. PathML [34] is another tool designed for machine learning applications in digital pathology and offers modules for stain normalization and foreground detection. PIMIP [35] offers an open source platform for pathology information management with WSI visualization, annotation, and image analysis. OpenHI2 [36] provides a user-friendly interface for histopathological image annotation, supporting slide viewing and region selection.

While a range of open-source tools support different stages of computational pathology workflows, each is typically designed with specific goals. Histolab, for instance, enables digital pathology preprocessing through capabilities such as tissue detection and artifact removal, along with automated testing features. However, its current scope does not extend to interactive annotation management. Tools like CellProfiler are well-established for analyzing small field-of-view microscopy images in biological studies but may require adaptation to apply to WSIs used in digital pathology. PathML is designed to support machine learning workflows with preprocessing features such as stain normalization and foreground detection, and may be used alongside other tools to complete downstream steps. Alongside these complementary efforts, HistomicsTK provides a more modular and extensible platform for comprehensive analysis of large-scale digital pathology images such as segmentation and quantitative feature extraction. Unlike some tools that are limited to small image fields, HistomicsTK offers a fully adaptable pipeline that is agnostic to image size and handles tiling and stitching, enabling the analysis of gigapixel WSIs.

Another crucial factor in digital pathology is the customizability and flexibility of tools, as user needs can vary significantly across research and clinical applications. Commercial tools, while effective and may be better optimized when specific or even proprietary information about the imaging device is taken into account, often lack these qualities. Their closed-source nature, high costs, and additional fees for expanded storage or processing capabilities can limit their adaptability to specific user requirements. In addition, digital pathology datasets are extremely large, making them difficult to analyze on local machines and challenging to transfer between systems. Centralized platforms that support seamless viewing, annotation, and analysis without the need to move or download large image files offer a major advantage. Furthermore, existing tools should ensure transparency and interpretability, as AI algorithms are often seen as “black boxes”; researchers require clear in-sights into underlying processes to ensure trust, reproducibility, and reliability. To address these challenges, we introduce HistomicsTK as an open-source, scalable, and customizable toolkit for digital pathology analysis, which will be explained in the following sections.

## 2. Software Description

### 2.1. Software architecture and functionality

**Fig. 1.**
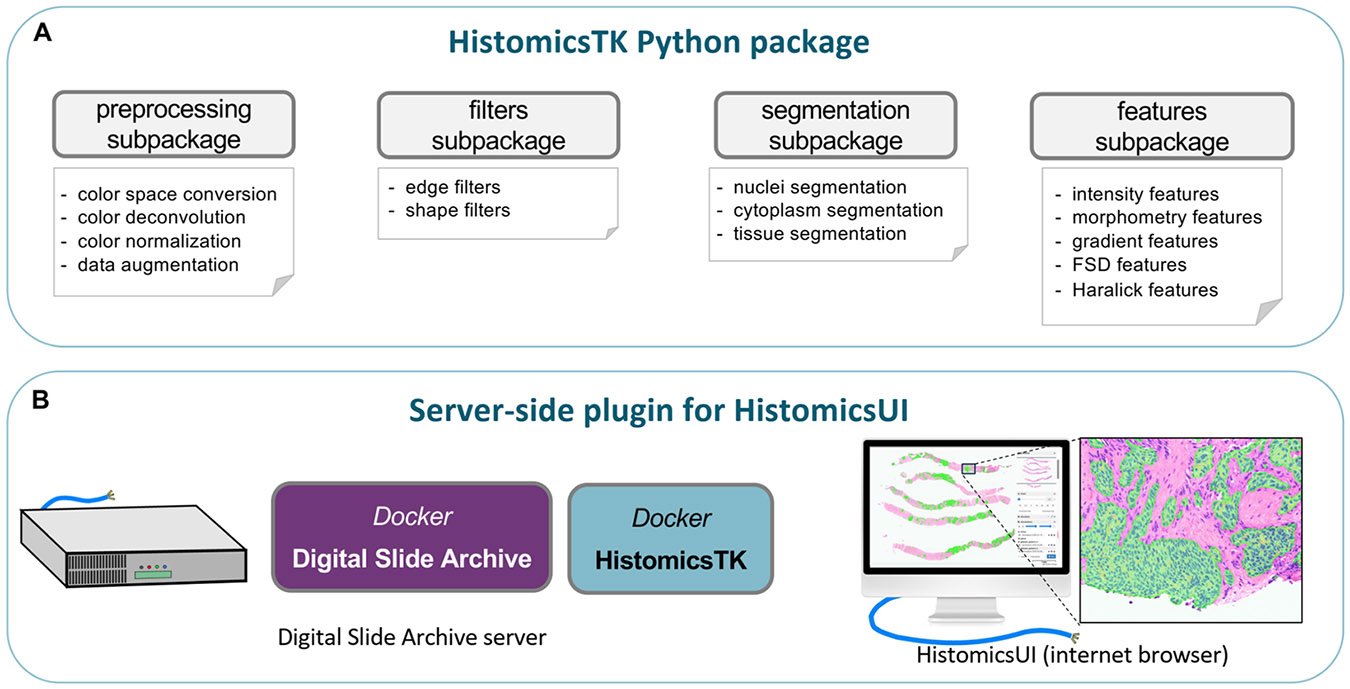
HistomicsTK can be used as a standalone python package (A) or as a server-side plugin (B). (A) HistomicsTK provides functions for preprocessing and filtering, image segmentation, and feature extraction that can be used to build histology image analysis pipelines. (B) As a server-side plugin, HistomicsTK integrates with the Digital Slide Archive to provide image analysis capabilities for hosted datasets. Users can invoke image analysis pipelines from their web browser and visualize outputs.

HistomicsTK can be deployed as a standalone Python package for building image analysis pipelines, or as a server-side plugin for the Digital Slide Archive (DSA), a web-based data management and visualization platform (see Fig. 1). As a standalone package, HistomicsTK is organized into five subpackages: Preprocessing, Filters, Segmentation, Feature extraction, and command-line interface (CLI). The Core subpackages of HistomicsTK, built using NumPy [37], scikit-image [38], SciPy [39], OpenCV [40], and scikit-learn [41] for numerical and image analysis and machine learning, are reviewed in detail below. Cython is used to accelerate bottlenecks in operations like boundary tracing and loop-heavy code with intensive array accessing. HistomicsTK is pip-installable with a matrix of wheels published on PyPI. A docker container is also provided on DockerHub that encapsulates package dependencies.

HistomicsTK is part of a software ecosystem for the management, visualization, and analysis of whole-slide digital pathology imaging data. This ecosystem includes the *large-image* package [43], that utilizes 16 different tile sources to support hundreds of file formats, including Digital Imaging and Communications in Medicine (DICOM) and popular proprietary formats, the *Digital Slide Archive*, an enterprise research platform for server-based management and visualization of digital pathology datasets, and *HistomicsUI*, a web-based user interface for image annotation and analysis. The CLI component implements end-to-end pipelines that can be invoked through HistomicsUI. This integration uses a 3D Slicer execution model (*slicer_cli_web*) that describes pipeline inputs and outputs via an XML specification, and that allows containerized algorithms to be hosted on DSA and exposed through HistomicsTK. The XML defines the interface for each CLI, enabling automated service discovery and UI generation, while a JSON file is used to specify environment configuration. The CLI component utilizes dask [44], a flexible parallel computing library, for parallel execution of CLIs. Dependencies are handled implicitly through the use of input/output files and explicitly through the pipeline orchestration engine including dask task graphs. Table 1 summarizes the architecture and key capabilities of HistomicsTK in comparison with QuPath [42].

### 2.2. Preprocessing

The preprocessing subpackage implements popular histology image preprocessing steps that transform or normalize images to improve downstream steps like segmentation or feature extraction. These include color space conversions, color deconvolution, color normalization, and stain augmentation. The color space conversion provides utility functions for transforming images between different color representations including optical density, stain darkness, red-green-blue, hue-saturation-intensity, and CIELAB. The color deconvolution enables the transformation of color images into stain intensity channels where the contributions of individual stains are isolated [45].Key functions include color_deconvolution, which performs the separation, and color_-convolution, which reconstructs color images from separated stains. For estimating the stain matrices used in deconvolution the rgb_separate_stains_macenko_pca and rgb_separate_stains_xu_snmf implement the Macenko [46] and sparse non-negative matrix factorization (SNMF) [47] methods, respectively.

Color normalization standardizes color profiles to minimize non-biological color variations and improve color consistency for subsequent processing steps. This capability is particularly critical in pathology, where discrepancies in tissue processing and imaging equipment can introduce significant color variability. This function offers two widely used stain normalization methods in digital pathology: deconvolution-based normalization [46] and Reinhard [48] normalization. These are implemented by the functions deconvolution_based_normalization and color_normalization.reinhard, which transform the color characteristics of an image to a desired standard. Color normalization largely depends on the color statistics of an ideal target image, and the color statistics of an input image. Independently normalizing different portions of a WSI, e.g. different tiles in a mosaic tiling, can produce localized color differences and strong boundary effects. To mitigate this, color_normalization.reinhard_stats can be used to calculate global slide-level color statistics for Reinhard color normalization. HistomicsTK also benefits from International Color Consortium (ICC) profile correction, which is supported through the use of large_-image for image formats that store this information [49].

The color augmentation can be used to generate augmented images with plausible color characteristics. The augmentation.perturb_stain_concentration function implements the method proposed by Tellez et al. [50] that augments stain concentrations in the staining darkness space prior to convolving them back into red-green-blue images.

### 2.3. Feature extraction

The feature extraction subpackage enables the extraction of quantitative features describing the shape, texture, and pixel intensity of segmented objects like cell nuclei.

features.compute_intensity_features extracts statistical features of pixel intensity values including the Minimum, Maximum, Mean, Median, Difference between mean and median, Standard deviation, Inter-quartile range, Median absolute deviation, Skewness, Kurtosis, and histogram-based Entropy and Energy features [51]. This can be applied to deconvolved stain intensity images to measure differences in staining intensities.

features.compute_morphometry_features computes morphometric properties of objects, including Orientation, Area, Convex hull area, Major and Minor axis lengths, Perimeter, Circularity, Eccentricity, Equivalent diameter, extent, Fractal dimension, Minor and Major axis ratio, Solidity, Hu moments [52], and weighted Hu moments, which offer invariant shape descriptors.

features.compute_gradient_features computes intensity gradient features by measuring the Mean, Standard deviation, Skewness, Kurtosis of gradient data, Entropy and Energy of the gradient magnitude histogram of object pixels, and Sum and Mean of canny filtered gradient data from an intensity image [51].

features.compute_fsd_features extracts Fourier shape descriptors, which measure object boundary shape in the frequency domain. This quantifies shape information by computing spectral energy across multiple predefined frequency bins to capture boundary smoothness or complexity [53].

features.compute_haralick_features extracts Haralick texture features from gray-level co-occurrence matrices (GLCMs) [54], including measures of textural uniformity, intensity variation, pixel intensity dependency, smoothness, and homogeneity. It also computes features related to pixel intensity distribution and variability (Sum Average, Sum Variance, Sum Entropy, and Entropy), and statistical differences between pixel intensities (Difference Variance and Difference Entropy), along with the first and second Information Measures of Correlation to assess pixel intensity interdependence.

To simplify cellular feature analysis, features.compute_nuclei_features, invokes the full range of feature extractions on nuclei and surrounding cytoplasm, using deconvolved nucleus and cytoplasm intensity images (for example from hematoxylin and eosin). Global features of cell locations can be calculated using features.compute_global_cell_-graph_features which uses Voronoi tesselations, Delaunay triangulation, and minimum spanning trees to describe spatial distributions of cells [55].

### 2.4. Filters

The filters subpackage offers a set of transformation functions designed to enhance various structures in histopathological images such as nucleoli or collagen fibers to aid in segmentation or feature extraction. The filters.edge includes methods such as edge.gaussian_grad, which performs smoothing using a derivative Gaussian kernel and leverages separable convolutions for efficient edge enhancement. The filters.shape offers advanced shape detection techniques, including shape.cdog, a scale-adaptive multi-scale difference-of-gaussian (DoG) filter for nuclei detection, inspired by scale invariant feature transform (SIFT)-based interest point detection [56]. Additionally, shape.clog function utilizes a constrained laplacian of gaussian (LoG) filter for nuclear seeding [57]. This subpackage also implements shape.glog, a generalized LoG filter for blob detection [58], and shape.vesselness, which measures filament and fiber-like structures using eigenvalues [59].

### 2.5. Segmentation

The segmentation subpackage provides segmentation methods and utilities for segmenting objects or building segmentation algorithms. Nuclei segmentation is provided by segmentation.nuclear, which includes algorithms such as Kofahi’s method based on multi-scale LoG filtering [57], gradient-field tracking [60], local maximum clustering [61], and Gaussian kernel voting for localizing nuclear centroids [62]. Functions for post-processing label images generated by segmentation are provided by segmentation.label, and can be used to trace object boundaries [63], and to clean label images removing small objects, spurs, and objects touching image boundaries. segmentation.rag also provides functions for label image processing to identify clusters of touching objects by graph-cuts, and to partition touching objects for parallel processing using graph coloring algorithms. Basic tissue segmentation in brightfield images is performed using a two-component Gaussian mixture model with kernel-density estimation (segmentation. simple_mask) for distinguishing tissue from background. For immuno-histochemically stained images, segmentation.positive_pixel_count enables the quantification of positive staining pixels for either individual fields (count_image) and entire slides (count_slide).

### 2.6. Command-Line interface

**Fig. 2.**
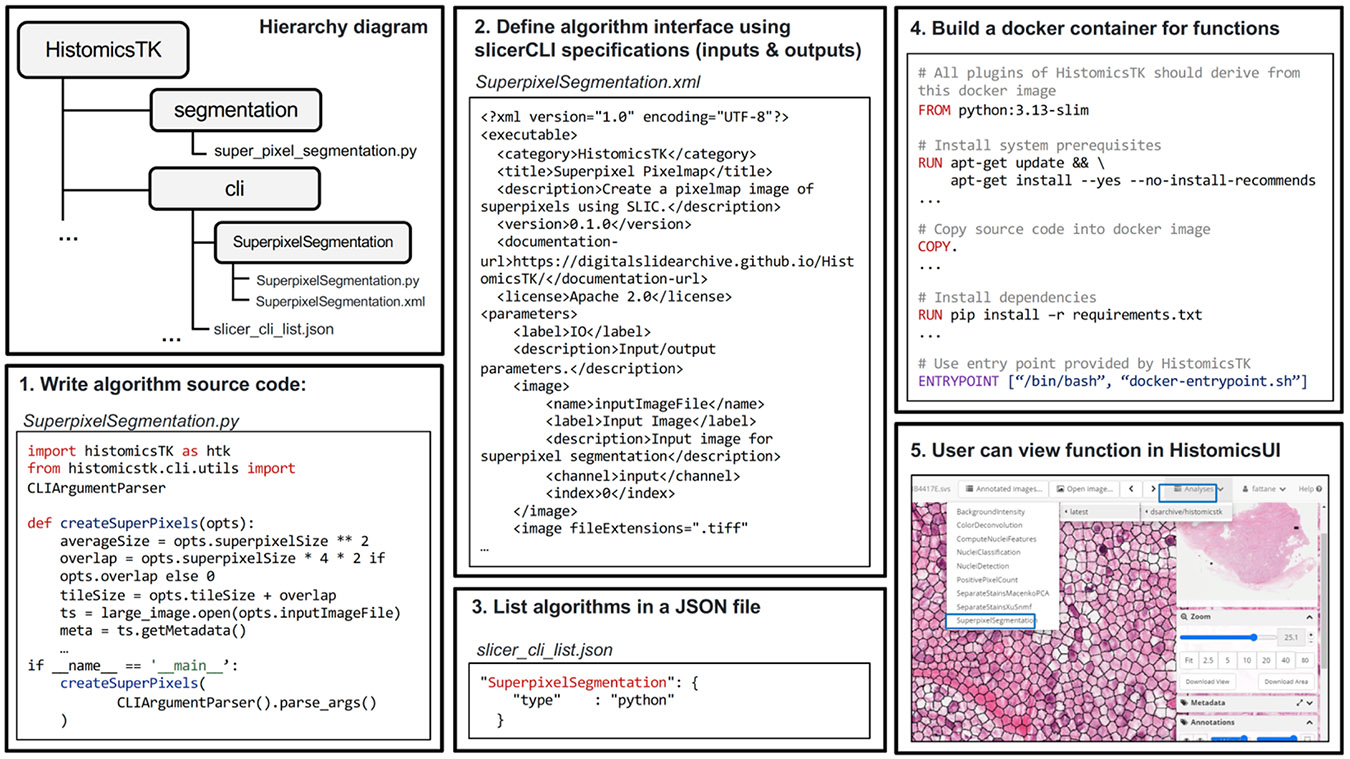
Command-line interface (CLI) framework in HistomicsTK. The toolkit facilitates the development and integration of modular image analysis algorithms via a standardized CLI framework. (1) Example algorithm source code written in Python, utilizing HistomicsTK’s core utilities such as CLIArgumentParser. (2) CLI specifications defined using the Slicer CLI XML schema, detailing inputs, outputs, and parameters. (3) Algorithms registered in a JSON configuration file for system-level discovery. (4) Dockerization of the package, including defined algorithms and dependencies. (5) End users execute the algorithms through the HistomicsUI web interface.

This subpackage contains CLIs that implement a range of image analysis pipelines and operations, including those found in other subpackages. Each CLI contains a python file that implements the operations, from parsing user-provided input argument, to image reading, invocation of image analysis functions, updating the console, and formatting outputs. This python file is accompanied by an XML file formatted in the 3D Slicer Execution Model Schema [64], which is used exclusively to define the names, types, and descriptions of pipeline inputs and outputs. This schema is required by the slicer_cli_web framework, which leverages the XML file to generate web-based user interfaces and configure execution on the DSA platform. The XML is not used for processing or visualization and does not imply a dependency on the 3D Slicer application itself. The parameters can be grouped to improve readability for end users (see Fig. 2). The XML definition also specifies expected outputs, which enables automated service discovery and output management in HistomicsUI.

CLIs implemented include nuclear detection and segmentation, feature extraction, classification, positive pixel count, and superpixel segmentation. Our approach to CLIs simplifies the deployment of pipelines to end users. Developers can implement their own pipelines with HistomicsTK and other tools and to serve these on the DSA platform. As a server-based solution, DSA can connect users with cluster computing resources to execute these pipelines on large hosted datasets.

## 3. Illustrative examples

**Fig. 3.**
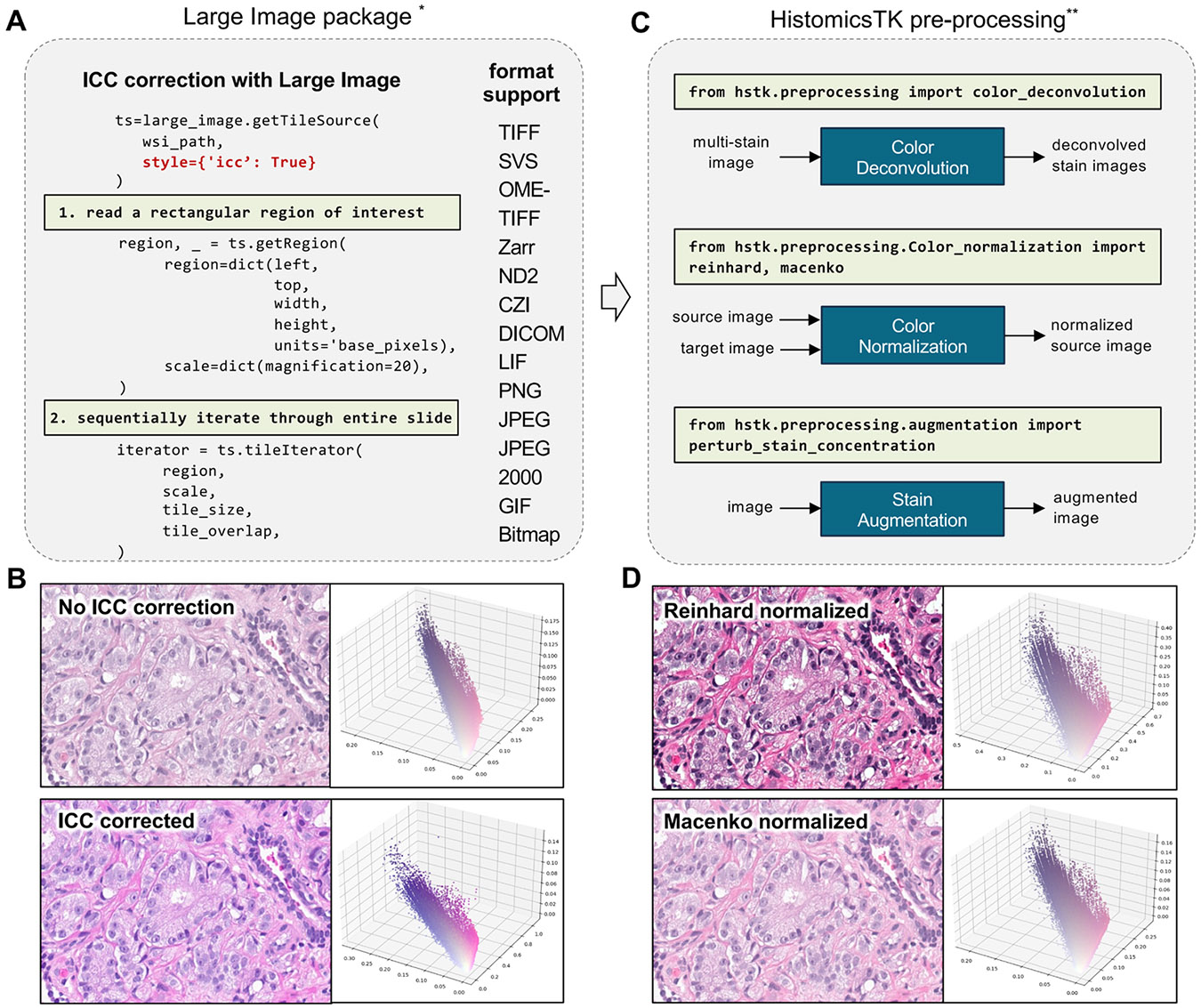
Reading and preprocessing images with HistomicsTK. (A) Large Image is a python package for reading WSIs that supports many of the proprietary file formats used in digital pathology. By default, Large Image applies ICC profile adjustments when available, but this can be disabled if needed. ICC correction can have a significant impact on the downstream performance of image analysis algorithms. (B) An example image with and without correction, with the pixel color distributions shown in RGB space. (C) HistomicsTK provides functions for color deconvolution, color normalization. And stain augmentation. (D) The field from (B) normalized with two different color normalization methods. * https://digitalslidearchive.github.io/HistomicsTK/examples/using_large_image.html# ** https://digitalslidearchive.github.io/HistomicsTK/examples/color_normalization_and_augmentation.html

**Fig. 4.**
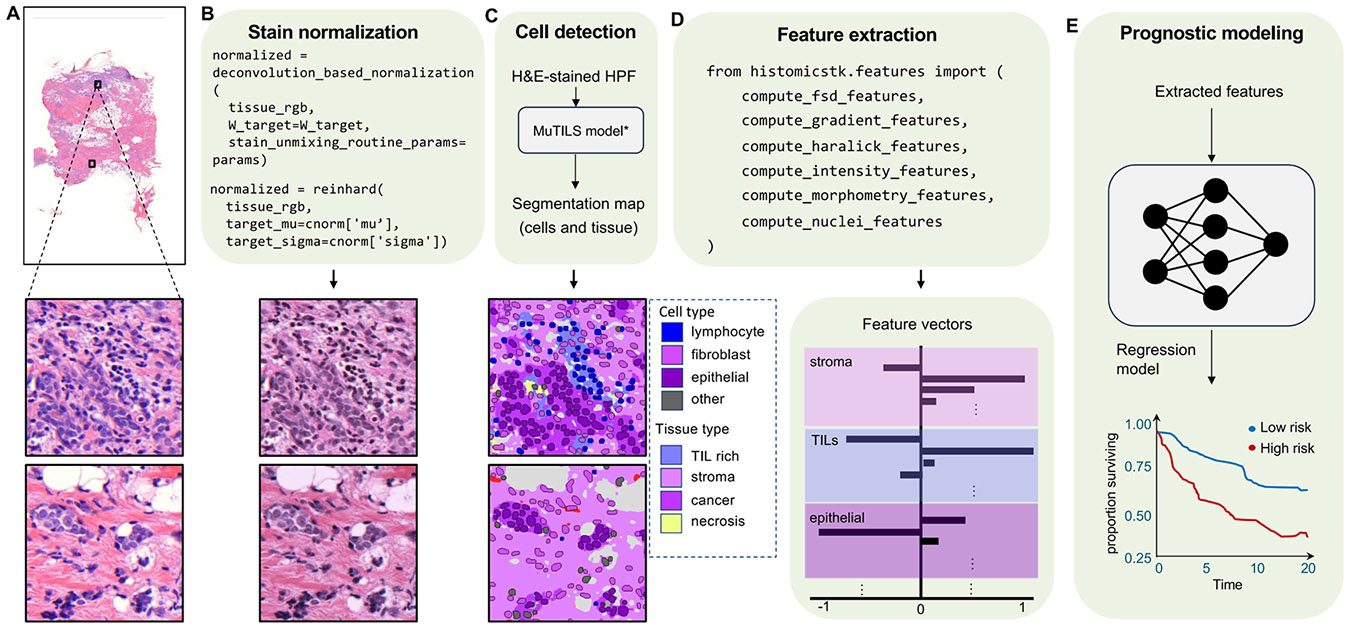
Example feature extraction workflow with HistomicsTK. (A) A WSI containing invasive breast cancer tissue. (B) Macenko color normalization is applied to correct color variability arising from staining and tissue processing. (C) The user applies a segmentation model to segment cells and tissue regions. (D) Feature extraction routines are used to describe the morphology of segmented objects, generating a quantitative representation of the slide. (E) Extracted features are used for tasks like prediction of prognosis. * External segmentation model (not in HistomicsTK)

Figs. 3 and 4 present illustrative workflows in which HistomicsTK and supporting packages are utilized for image preprocessing, and feature extraction in digital pathology. Fig. 3A, illustrates WSI file formats supported by Large Image package along with basic image reading functionalities. ICC profiles are applied to images when available, allowing for the correction of color inconsistencies introduced during acquisition.

The impact of ICC correction is demonstrated in Fig. 3B, where a representative region is shown before and after correction, along with the respective RGB pixel distributions. Subsequently, preprocessing operations including color deconvolution, normalization, and stain augmentation are carried out using subpackages provided by HistomicsTK, as outlined in Fig. 3C. The results of applying two commonly used color normalization methods, Reinhard and Macenko, are visualized in Fig. 3D, where the same image region is shown with improved color consistency.

In Fig. 4, a representative feature extraction workflow using HistomicsTK is illustrated. A WSI containing invasive breast cancer tissue is used as input (Fig. 4A), and stain normalization is performed using the Macenko method to mitigate color variability due to differences in staining protocols (Fig. 4B). A segmentation model is then applied to high-power fields (HPFs) to delineate cells and tissue compartments, producing a segmentation map (Fig. 4C). Following segmentation, various morphological, textural, and intensity-based features are computed from the segmented objects using HistomicsTK’s feature extraction (Fig. 4D). These features are used to generate quantitative representations of various tissue types, such as stroma, TILs, and epithelial regions. Finally, in Fig. 4E, the extracted features are integrated into a prognostic modeling framework, where they are utilized for tasks such as patient risk stratification based on survival outcomes. Through these examples, the modularity and reproducibility of image analysis workflows enabled by HistomicsTK are demonstrated.

We evaluated pixel-wise performance of nuclei segmentation algorithms in HistomicsTK using the Panoptic segmentation of nUclei and tissue in advanced MelanomA (PUMA) [65] dataset, and the PanNuke [66] dataset, which provides pan-cancer histology images for nuclei instance segmentation and classification. The PUMA dataset comprises H&E-stained tiles from 205 melanoma specimens (103 primary and 102 metastatic). Each tile, selected at 40× magnification (0.23 μm/pixel), has a resolution of 1024×1024 pixels. Ground truth annotations for this dataset were initially generated semi-automatically, and subsequently verified and corrected by a dermatopathologist. In total, the PUMA dataset contains 85,715 annotated cell nuclei. The PanNuke dataset includes 2656 H&E-stained tiles extracted from WSIs, each with a resolution of 224×224 pixels and acquired at 40× magnification. The dataset contains 53,755 annotated cell nuclei, which encompasses 19 different tissue types, semi-automatically annotated and quality-controlled by clinical pathologists.

**Fig. 5.**
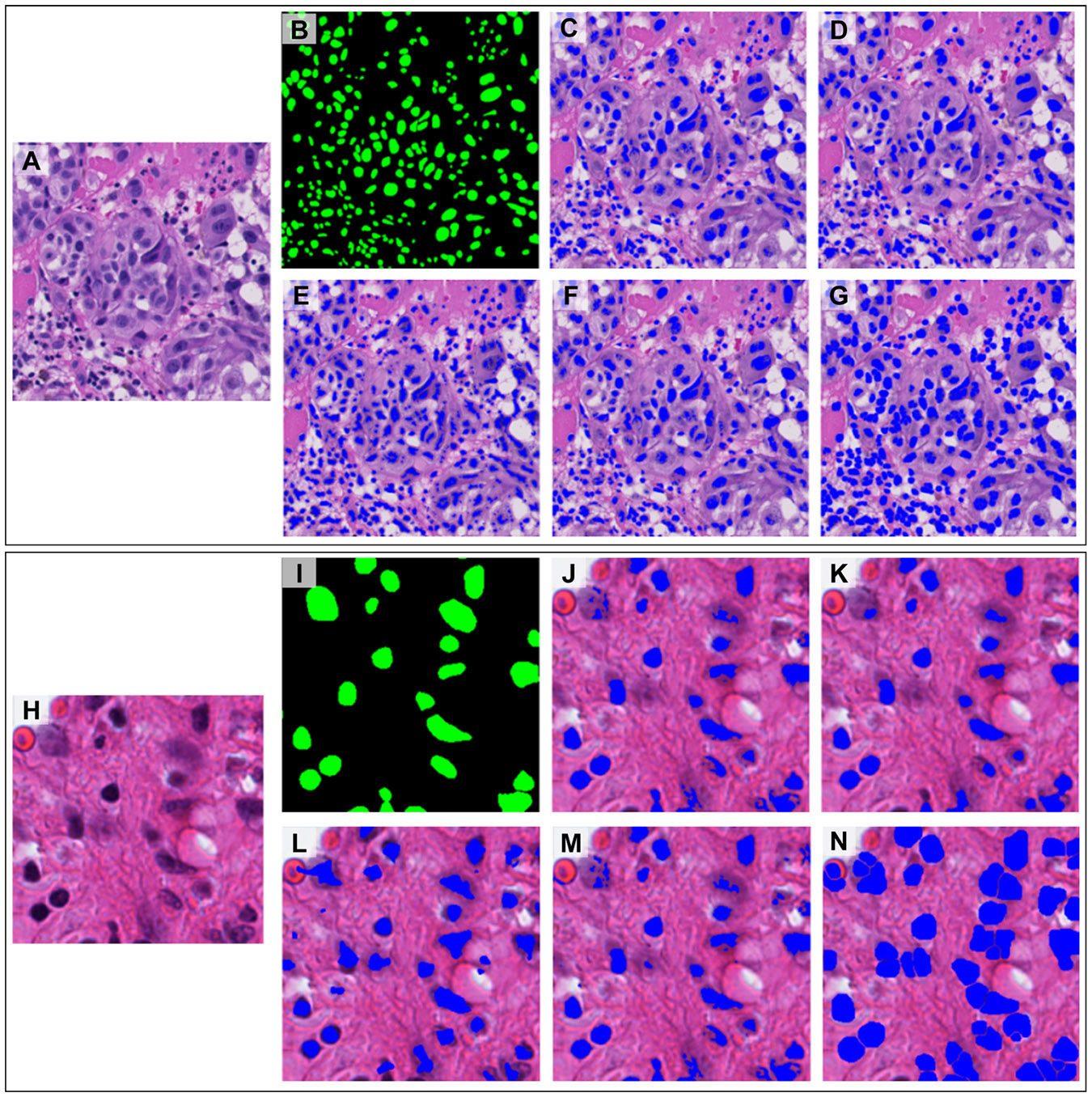
Visual comparison of nuclei segmentation results using HistomicsTK and QuPath [42] on representative tiles, shown alongside corresponding ground truth annotations. Sample tiles of size 1024 × 1024 and 224 × 224 pixels from the PUMA [65] and PanNuke [66] datasets are shown in (A, H), respectively, with ground truth nuclei annotations in (B, I) and segmentation results for the local maximum clustering algorithm (C, J), Kofahi’s method with multi-scale LoG filtering (D, K), the Gaussian kernel voting approach (E, L), the gradient-field tracking algorithm (F, M), and QuPath’s Watershed-based method (G, N).

Table 2 summarizes the comparison of various nuclei segmentation algorithms available in HistomicsTK and QuPath [42], in terms of computational performance (runtime and memory usage) and pixel-wise segmentation accuracy. The reported metrics include precision, recall, accuracy, F1 score, Intersection over Union (IoU), runtime in seconds, and peak memory usage in megabytes. Fig. 5 illustrates the performance of these algorithms applied to representative tiles from the PUMA and PanNuke datasets, in comparison with the corresponding ground truth. All experiments were run on a server equipped with an Intel^®^ Xeon^®^ Gold 6226R 64-core CPU running at 2.90 GHz and 377 GB of RAM.

## 4. Impact

Microscopic examination of tissue patterns using glass slides is a time-honored practice, and advances in imaging devices, storage, and computing now enable quantitative and reproducible evaluation of these patterns by analysis of digital images. Extracting information from histology images requires developing pipelines that address challenges in efficient loading of data stored in a wide array of proprietary formats, reducing variability arising from tissue processing and imaging, delineating structures of interest, and measuring their morphologic properties. Commercial solutions for digital pathology are often cost-prohibitive in academic research and typically lack the flexibility required for customization to specific research needs.

HistomicsTK provides core image analysis capabilities in an open-source pip-installable Python package that is extensible and customizable. Among open-source tools for digital pathology, HistomicsTK fills gaps in preprocessing and feature extraction, providing access to a large number of image transformation and normalization methods, and a comprehensive set of image features that are designed for characterizing histology structures. Notably, the library is specifically designed to process arbitrarily large image files, making it suitable for processing whole slide images.

Putting image analysis capabilities in the hands of biomedical researchers has been a major challenge in this field. Thick clients like QuPath [42] that provide image analysis capabilities are limited to the storage and computing power of desktop or notebook machines, where WSI datasets often contain hundreds of terabytes of digital images. Through integration with DSA, a server-based platform, HistomicsTK enables users to execute image analysis pipelines on large hosted datasets using high-performance computing clusters. The containerization framework used for this integration is also accessible to developers to package and host their own image analysis pipelines. The core operations of HistomicsTK are highly optimized using a combination of vectorization and Cython acceleration of bottlenecks. HistomicsTK uses Cython for a few of its algorithms to substantially improve their performance. Cython is applied only when an algorithm contains tight loops that are not amenable to vectorization using NumPy. One benefit is that we can use the algorithms in the forms in which they were originally published, so that we know we are implementing them as intended rather than creating a similar algorithm that uses different techniques. An example of one such algorithm is tracing object boundaries with Moore neighborhoods. Dask is also utilized in many of the included pipelines to parallelize the processing of image regions.

HistomicsTK has been widely adopted, having been downloaded over 49,460 times from PyPI [67] between March and April 2025. It has been cited in over 150 peer-reviewed publications including the exploration of digital biomarkers for invasive breast cancer [14], classification and segmentation of nuclei in breast cancer specimens [15], quantitative analysis of tumor-infiltrating lymphocyte [9,10], and extraction of pathomic features from H&E stained images to predict the expression of protein biomarkers [11]. Similar to the Insight Toolkit (ITK) [68], HistomicsTK operates under a community development model that encourages community participation in software development and maintenance. It has contributions from 32 developers, and over 434 stars and 122 forks on GitHub [69].

## 5. Conclusion

The rapid growth of digital pathology data is creating new opportunities to improve understanding of complex diseases. Open-source software can play an important role in realizing this goal, by empowering researchers to extract information from histology images in a reproducible manner. HistomicsTK is a pip-installable Python package that provides algorithm developers with building blocks to create complex image analysis pipelines. HistomicsTK also provides packaged pipelines that can be served to end users through a web-based interface using an integration mechanism that also allows developers to containerize and serve their own pipelines. Similar to other image analysis tools like ITK and VTK [70], HistomicsTK encourages community participation in development and maintenance, and uses continuous integration to encourage participation while maintaining package quality. While HistomicsTK represents one of several available solutions for histology image analysis, its emphasis on preprocessing and feature extraction fills important gaps in the open-source tool domain, and it offers researchers an easy-to-deploy and extensible alternative to commercial software. Future developments will focus on incorporating newly published methods, improving integration with deep learning frameworks, and enabling graphical processing acceleration for additional modules.

## Figures and Tables

**Table 1:** Comparison of HistomicsTK and QuPath [42]: architecture and key capabilities.

Module	HistomicsTK	QuPath
Deployment	- Standalone Python package or server plugin for DSA - Web-based UI via HistomicsUI - Docker + PyPI support	- Desktop application - Can execute jobs on clusters, but not designed as server-based software
User Interface	- Web-based through DSA and HistomicsUI	- Desktop GUI with interactive tools
Core Stack	- NumPy, SciPy, scikit-image, scikit-learn, OpenCV - Accelerated with Cython	- Java, JavaFX, Groovy - OpenCV (via JavaCPP) - Other Java libraries for image processing
Preprocessing	- Color conversions: RGB, optical density, HSI, CIELAB - Stain separation: Macenko, SNMF - Normalization: Reinhard, deconvolution -based - Augmentation: stain perturbation	- Stain separation (color deconvolution) - Stain vector estimation
Segmentation	- Nuclei segmentation: Kofahi’s method based on multi-scale LoG filtering, gradient-field tracking, local maximum clustering, Gaussian kernel voting - superpixel segmentation - Label refinement: cleaning, graph cuts - Tissue mask + positive pixel count	- Cell detection: Watershed, Difference of Gaussians (DoG) - Pixel classification
Feature Extraction	- Intensity and histogram based features - Morphometry features - Haralick texture features - Gradient features - Fourier shape descriptors - Graph based Voronoi, Delaunay, and minimum spanning trees features	- Intensity - Shape - Morphometry - Haralick texture features - Delaunay
Filters	- Edge filters: Gaussian gradient - Shape filters: Difference-of-Gaussian (DoG), Laplacian of Gaussian (LoG), generalized LoG, vesselness	- Gaussian blur - Morphological filters - Laplacian of Gaussian
Command-Line Interface/Pipelines	- Exposed via slicer_cli_web Including CLIs for nuclear detection and segmentation, feature extraction, classification, positive pixel count, superpixel segmentation - Supports parallel execution with dask	- Scripting capabilities via command line

**Table 2:** Pixel-wise segmentation accuracy and computational performance of nuclei segmentation algorithms in HistomicsTK and QuPath on the PUMA [65] and the PanNuke [66] H&E-stained datasets. The evaluated algorithms include Kofahi’s method based on multi-scale LoG filtering [57], gradient-field tracking [60], local maximum clustering [61], Gaussian kernel voting [62], and QuPath’s Watershed cell detection [42].

Data	Segmentation algorithm	precision (std) %	recall (std) %	accuracy (std) %	F1 score (std) %	IoU (std) %	runtime (std) s	peak memory usage (std) MB
PUMA [65]	htk.segmentation.nuclear .	81.47	52.14	86.77	62.29	46.37	0.32	31.63
	max_clustering	(±16.11)	(±14.12)	(±05.64)	(±12.84)	(±12.35)	(±0.04)	(±0.47)
	htk.segmentation.nuclear .	82.55	52.03	86.88	62.43	46.66	1.10	121.01
	detect_nuclei_kofahi	(±16.13)	(±14.96)	(±05.89)	(±13.60)	(±13.08)	(±0.07)	(±0.00)
	htk.segmentation.nuclear .	68.65	56.47	84.96	60.99	44.24	19.15	53.08
	gaussian_voting	(±12.96)	(±08.03)	(±04.00)	(±07.32)	(±07.08)	(±2.12)	(±1.26)
	htk.segmentation.nuclear .	79.55	55.84	87.06	64.36	48.49	44.87	216.93
	gvf_tracking	(±16.04)	(±13.60)	(±05.32)	(±12.10)	(±11.84)	(±12.15)	(±53.22)
	QuPath [42]	49.95	83.22	78.27	61.34	44.66	6.55	762
	(WatershedCellDetection)	(±09.88)	(±10.11)	(±05.23)	(±07.66)	(±07.71)	(±16.12)	(±53.94)
PanNuke [66]	htk.segmentation.nuclear .	79.36	52.72	87.24	58.72	43.48	0.01	1.94
	max_clustering	(±19.51)	(±20.72)	(±12.29)	(±16.91)	(±16.23)	(±0.00)	(±0.04)
	htk.segmentation.nuclear .	81.91	51.88	87.35	58.94	44.08	0.05	7.57
	detect_nuclei_kofahi	(±17.98)	(±22.20)	(±12.65)	(±18.46)	(±17.76)	(±0.00)	(±0.00)
	htk.segmentation.nuclear .	58.80	44.03	83.54	46.67	31.35	0.99	3.47
	gaussian_voting	(±21.21)	(±13.15)	(±11.42)	(±13.30)	(±10.50)	(±0.18)	(±0.12)
	htk.segmentation.nuclear .	75.51	56.82	87.30	60.00	44.60	1.38	14.80
	gvf_tracking	(±20.92)	(±19.44)	(±11.62)	(±16.05)	(±15.36)	(±0.64)	(±6.96)
	QuPath [42]	55.05	68.72	82.28	54.78	39.43	4.25	412
	(WatershedCellDetection)	(±16.64)	(±26.25)	(±12.54)	(±17.11)	(±14.63)	(±0.07)	(±16.28)
